# The Effect of Growth-Mimicking Continuous Strain on the Early Stages of Skeletal Development in Micromass Culture

**DOI:** 10.1371/journal.pone.0124948

**Published:** 2015-04-27

**Authors:** Darinka D. Klumpers, Theo H. Smit, David J. Mooney

**Affiliations:** 1 School of Engineering and Applied Sciences, Harvard University, 29 Oxford St, Cambridge, Massachusetts, 02138, United States of America; 2 Wyss Institute for Biologically Inspired Engineering, Harvard University, 3 Blackfan Circle, Boston, Massachusetts 02115, United States of America; 3 Dept. Orthopedic Surgery, Research Institute MOVE, VU University Medical Center, De Boelelaan 1117, 1081 HV, Amsterdam, The Netherlands; National Institutes of Health, UNITED STATES

## Abstract

Embryonic skeletogenesis involves proliferation, condensation and subsequent chondrogenic differentiation of mesenchymal precursor cells, and the strains and stresses inherent to these processes have been hypothesized to influence skeletal development. The aim of this study was to determine the effect of growth-mimicking strain on the process of early skeletal development *in vitro*. To this end, we applied continuous uniaxial strain to embryonic skeletal precursor cells in micromass culture. Strain was applied at different times of culture to specifically address the effect of mechanical loading on the sequential stages of cellular proliferation, condensation and differentiation. We found that growth-mimicking strain at all three times did not affect proliferation or chondrogenic differentiation under the tested conditions. However, the timing of the applied strain did play a role in the density of mesenchymal condensations. This finding suggests that a mechanically dynamic environment, and specifically strain, can influence skeletal patterning. The growth-mimicking micromass model presented here may be a useful tool for further studies into the role of mechanical loading in early skeletal development.

## Introduction

During early embryonic development, the skeletal elements arise through a process of proliferation, mesenchymal condensation and subsequent chondrogenic differentiation of skeletal precursor cells. These processes take place in a highly dynamic environment, both chemically and physically. Secreted morphogens create spatial and temporal signaling gradients, which have been shown to play a role in the determination of skeletal patterns and morphogenesis in general [1,2]. The role of the mechanical environment however, and its temporal and spatial dynamics, are less well understood. The observation that neuromuscular disorders, which cause reduced muscle contraction in the developing embryo, lead to abnormal skeletal structures provides evidence of the effect of forces on skeletal development [3]. In earlier stages, at the initiation of skeletal development, cell generated traction and overall growth of distinctive tissues result in tissue-level stresses and strains [4]. These mechanical cues are hypothesized to play an important role in skeletal development [4,5], but well-controlled experiments are required to provide insight into this process.

Various cell culture models, including the micromass assay, have been developed to study early skeletal development *in vitro* [6–9]. In the micromass assay, pre-chondrogenic cells are typically isolated from chicken or mouse embryonic limb buds in the pre-condensation phase and plated in a high-density drop on a culture dish [9]. After adhering to the dish, the cells proliferate while depositing abundant extracellular matrix, mimicking the proliferation stage taking place within the population of mesenchymal stem cells during the first stage of embryonic skeletogenesis [10]. Within two days, when a critical cell density is reached, mesenchymal condensations appear in the micromass culture, characterized by a locally increased cell density and positive staining with peanut agglutinin lectin (PNA) [11]. This process mimics the formation of mesenchymal condensations during skeletal development, which determine the location and pattern of future skeletal elements [12]. Mesenchymal condensation is believed to occur through passive cell movements and cellular rearrangements rather than active cell migration or localized proliferation [13,14]. During skeletogenesis, the increased cell density in condensations is accompanied by an increase in cell-cell contacts mediated through NCAM and N-cadherin, which are thought to induce chondrogenic differentiation [15,16]. Similarly, in the micromass culture chondrogenic differentiation is initiated, as indicated by increased expression of chondrogenic genetic markers such as Sox9, and the deposition of glycosaminoglycans and collagen type II in the condensations [17]. At this point, multiple layers of cells are embedded in abundant ECM and the culture can thus be considered three-dimensional.

The micromass assay is typically performed on static, rigid culture plastic [9], but during early development, the embryo is growing rapidly. Different tissues grow and expand at different rates, while a wide range of cell types generate differential amounts of cellular traction forces [18,19]. This creates growth-induced pressures and strains, as well as direct deformation of the tissues [4]. Since the dynamic character of the environment is thought to affect early skeletal development, it could be revealing to incorporate such cues in the micromass assay in order to more fully recapitulate the *in vivo* process. Mechanical loading has been previously applied to micromass cultures, however the results vary widely [20,21]. Also, the loading conditions in those experiments were designed to mimic loading of adult cartilage rather than loading in the growing embryo.

In this study, we address the question whether growth-mimicking strain, or deformation, affects early skeletal development *in vitro* by subjecting the widely used micromass model to a slow continuous strain. Uniaxial continuous strain was applied during the first, middle or last part of the culture period, to specifically address the effect of mechanical loading on the respective stages of early skeletal development: proliferation, condensation and differentiation. It was found that, under the specific conditions tested, growth-mimicking strain at any time point did not affect proliferation or chondrogenic differentiation of the skeletal precursor cells; however, the timing of the applied strain did play a role in the density of mesenchymal condensations.

## Materials and Methods

### Cell isolation and culture

Pre-chondrogenic cells were freshly isolated from limb buds of Hamburger Hamilton stage 21–23 chicken embryos [22], as previously described [23]. Fertilized eggs (White Leghorn) were obtained from Charles River Laboratories (New York, NY). Dissected limb bud were incubated in 0.5% trypsin at room temperature for 11 min and transferred to ice-cold 10% chicken serum. The ectodermal layer was then removed manually. The buds were transferred to DMEM/F12 culture medium containing 10% FBS and 1% penicillin and streptomycin, pipetted up and down to create a single cell suspension, and strained through a 40μm filter. 6μl of a cell suspension of 2*10^7^ cells/ml was pipetted into each mini-well (described in the following section) and incubated for 1 hour. The wells were then flooded with 2.5ml DMEM/F12 containing 2% FBS and 1% penicillin and streptomycin. Culture medium was replaced after 24 hours, and the cultures were terminated at 60hrs, unless stated otherwise.

### Strain

**Fig 1:**
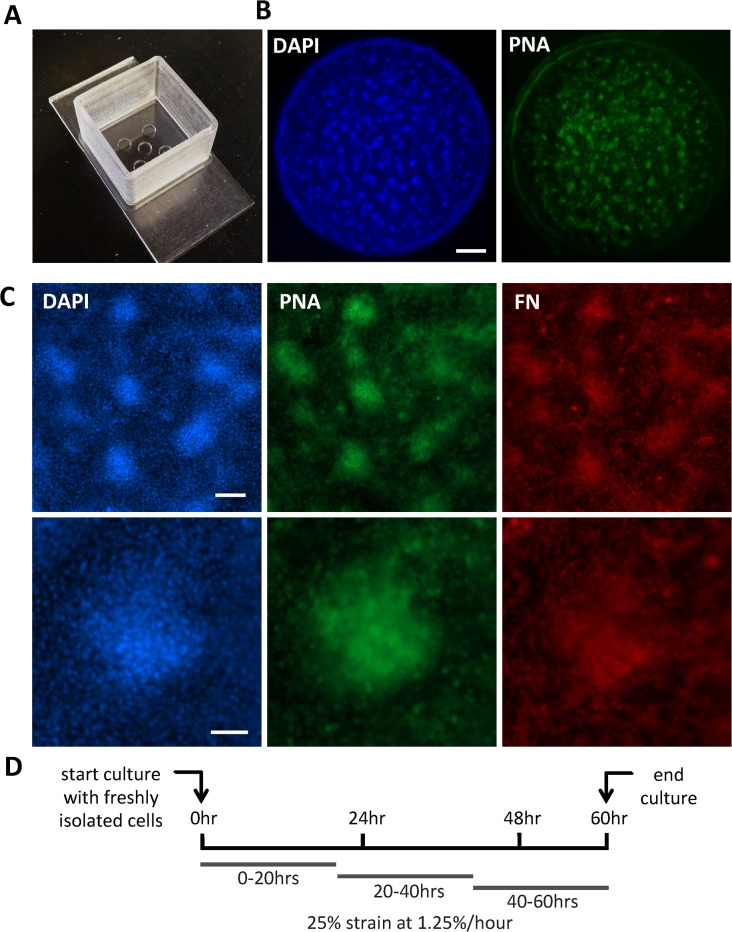
Experimental model. **(A)** Bird view of a PDMS well containing five mini-wells. **(B)** Fluorescent micrographs of a representative well cultured for 60 hours in the absence of strain, stained with DAPI to indicated nuclei (blue, left) and peanut agglutinin lectin (PNA) to indicate condensations (green, right). Scale bar is 500μm. **(C)** Fluorescent micrographs of representative cultures after 60 hours of culture in the absence of strain. Cells are stained with DAPI (blue), peanut agglutinin lectin (PNA, green), and an anti-avian fibronectin (FN) antibody to indicate deposited FN (red). Lower magnification (top row): scale bar is 100μm. Higher magnification (bottom row): scale bar is 50μm. **(D)** Schematic representation of the time line of the experiments. Cells are freshly isolated and directly plated onto the PDMS wells. Continuous strain is subsequently initiated at 0hr, 20hr, or 40hr, for a total of 25% strain over 20hours. Cultures are terminated at 60 hours.

Flexible polydimethylsiloxane (PDMS) wells were fabricated by mixing PDMS base and curing agent (Sylgard 184 silicon elastomer kit) in a 20:1 ratio, pouring into custom-made Teflon molds [24] and curing at 65°C overnight. The PDMS wells were designed to contain five mini-wells with a surface area of 10mm^2^ and a depth of 0.5mm (Fig 1A). The PDMS wells were subjected to O_2_ plasma treatment (Diener Electronic, Plasma Surface Technology) for 5 min to render the surface hydrophilic and coated with fibronectin (FN, 15μg/ml (bovine, Sigma) in dH_2_O) overnight at 4°C. Freshly isolated cells were seeded onto the mini-wells and incubated until the start of the strain. At the initiation of the strain regime, the wells were mounted onto a uniaxial stretch device [24]. Non-strained controls were placed in the device chamber as well to ensure identical culture conditions. Samples were then subjected to continuous uniaxial strain at a strain rate of 1.25% per hour for a total of 25% strain. The strain rate was chosen based on the overall growth rate of chicken embryos at this stage in development [22]. Strain was started either at 0hrs, 20hrs, or 40hrs of culture for a period of 20hrs and cultures were terminated at 60hrs (Fig 1D).

### Immunohistochemistry and staining

At the end of the experiment, cultures were fixed in 4% paraformaldehyde in PBS. To identify condensations, samples were stained with 50 μg/ml Alexa Fluor 488 conjugated peanut agglutinin lectin (Invitrogen) and counterstained with 2 μg/ml Hoechst 33342. To stain for deposited FN, samples were probed with a mouse-anti-avian FN antibody (Developmental Studies Hybridoma Bank) and stained with an Alexa Fluor 546 conjugated goat-anti-mouse IgG. Deposited glycosaminoglycans were visualized with Alcian Blue staining (1% Alcian Blue in 3% acetic acid (Electron Microscopy Sciences) for 30 minutes).

### Glycosaminoglycan and DNA quantification

The deposition of glycosaminoglycans was quantified using a dimethyl methylene blue (DMB) quantification method, and normalized to DNA content to yield GAG/DNA ratios. After 60hrs, whole cultures were digested in papain digestion buffer (0.125 mg/ml papain enzyme (Sigma), 10 mM L-cysteine, 100 mM sodium phosphate buffer, 10 mM Na_2_EDTA, pH 6.5) for 24 hours at 65°C. Directly following digestion, the DNA content was determined using a PicoGreen dsDNA assay kit (Invitrogen). GAG content was determined colorimetrically by mixing 10μl sample with 175μl DMB staining solution (2.37 mg/ml NaCl, 3.04 mg/ml glycine, 16 μg/ml 1,9-dimethyl methylene blue, pH 3) and directly reading the absorbance at 590 and 525nm in a BioTek Synergy HT plate reader.

### EdU proliferation assay

Proliferation was assessed at 48 hours by analyzing the incorporation of EdU over a 4-hour time window (44–48 hrs), using a Click-iT EdU Alexa Fluor 555 kit (Invitrogen). In order to quantify the percentage of EdU positive cells, the cells were trypsinized, fixed, permeabilized with 0.5% Triton X-100, stained for 30 minutes following the manufacturer’s protocol, and analyzed by flow cytometry. For microscopic imaging, whole cultures were fixed at 48hrs, stained for EdU and counterstained with 2 μg/ml Hoechst 33342 (Invitrogen).

### Flow cytometry

Flow analysis of fluorescently stained cells was performed on a BD LSR II Analyzer. To evaluate Sox9 expression, cells were trypsinized at 60hrs, fixed, permeabilized in 0.5% Triton-X-100, probed with a rabbit-anti-Sox9 polyclonal antibody (Millipore) at 5 μg/ml, and stained with an Alexa Fluor 647conjugated goat-anti-rabbit IgG (Invitrogen). The Mean Fluorescent Intensity (MFI) is reported as a measure of Sox9 expression.

### Quantification of density of condensations

The number of condensations was quantified using images of PNA staining, based on the known local increase in the intensity of this staining in the condensations. Two representative regions of interest in the center of each well were chosen to avoid edge effects, and condensations were identified using the ‘Find maxima’ function in ImageJ. The number of condensations was then normalized to the area of the chosen region to yield the density of condensations. For each condition, 5 independent micromass cultures were analyzed. Data are represented as means ± standard deviation.

### Statistical analysis

One-way ANOVA analyses were performed to test for statistical significance between the four experimental conditions for all output parameters tested. A significance level of 0.05 was used. In case the ANOVA analysis showed a significant difference between groups, a post-hoc Bonferroni multi-comparison test was performed to identify which experimental conditions were significantly different.

### Ethics Statement

The use of chicken embryos for this study was approved by the Institutional Animal Care and Use Committee (IACUC) of the Faculty of Arts and Sciences at Harvard University under protocol #28–13.

## Results

### Experimental model system

In order to address the question whether growth-mimicking strain influences the early processes of skeletal development *in vitro*, we adapted the classic micromass assay so that continuous strain could be applied to the live cultures. During the 60hr-culture, mesenchymal condensations formed all throughout the circular mini-well (Fig 1B). The formation of typical mesenchymal condensations was confirmed by the observation of a high local cell density, enhanced peanut agglutinin lectin staining, and abundant FN deposition (Fig 1C).

### Proliferation

**Fig 2:**
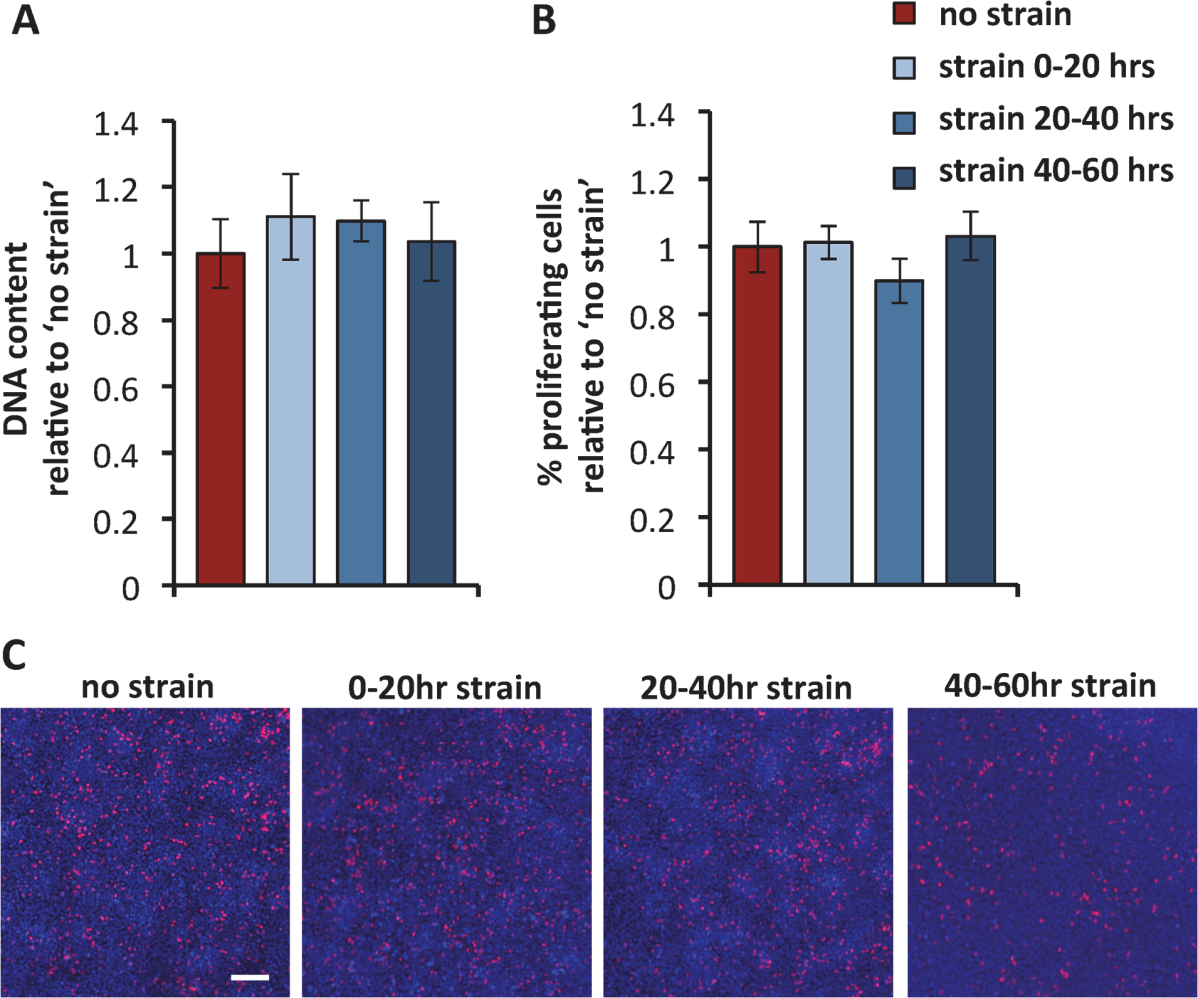
Proliferation. **(A)** Total DNA content is measured after 60 hrs. Data are normalized to the non-strained condition and represent means ± standard deviations, n≥8. **(B)** Percentages of proliferating cells, indicated by the incorporation of EdU, were measured at 48 hour of culture. Data are normalized to the non-strained condition and represent means ± standard deviations, n≥5. **(C)** Fluorescent micrographs of 48-hour cultures stained for nuclei (DAPI, blue) and EdU (red), after incubation with EdU for 4 hours. Scale bar is 100μm.

First, the effect of growth-mimicking strain on cell proliferation was assessed, because proliferation is an important first step in early skeletal development and micromass cultures [10,25]. As an indication of proliferation, the total DNA content was measured at the end of the culture at 60hrs. No significant differences in DNA content were observed between the non-strained condition and the variously timed regimes of 25% strain (Fig 2A). Proliferation was then analyzed more specifically by the incorporation of EdU at 48hrs. There were again no statistically significant differences in the percentage of EdU positive cells between the conditions (Fig 2B). Additionally, the spatial distribution of proliferating cells was similar for the non-strained and strained samples, with no discernable spatial patterning (Fig 2C).

### Mesenchymal condensation

**Fig 3:**
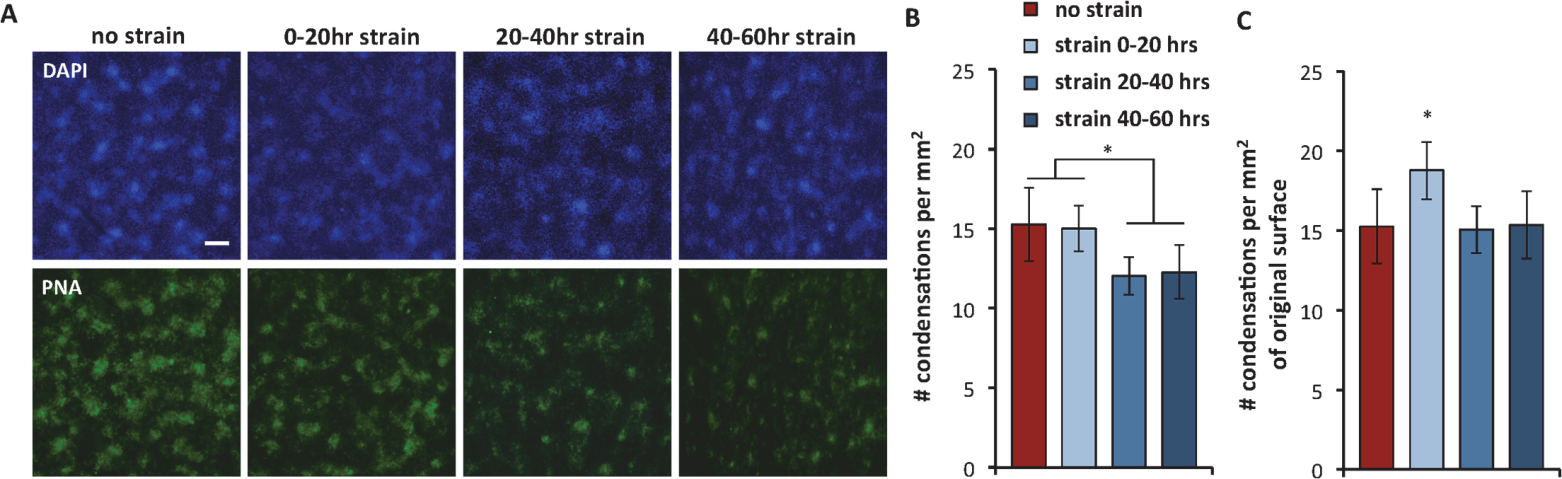
Mesenchymal condensation. **(A)** Fluorescent micrographs of samples cultured for 60 hours. Cultures are stained with DAPI (blue, top row) and peanut agglutinin lectin (PNA, green, bottom row). Scale bar is 200μm. **(B)** The number of condensations per squared mm is quantified at 60 hours, using images of PNA staining. Data represent means ± standard deviations, n≥7. *, p < 0.05. **(C)** The number of condensations per squared mm for the three strained conditions is corrected for the applied strain, thus multiplied by a factor of 1.25. The graph displays the number of condensations per squared mm of original surface area. Data represent means ± standard deviations, n≥7. *, p < 0.05.

Next, we analyzed the formation of mesenchymal condensations under the various culture conditions. Condensations, indicated by increased cell densities and enhanced peanut agglutinin lectin staining, appeared morphologically similar across all tested conditions (Fig 3A). Subsequently, we quantified the density of condensations as a function of strain (Fig 3B). While the density of condensations was comparable for the non-strained condition and the 0–20hrs strain regime, the densities for the 20–40hrs and the 40–60hrs strain regime were significantly lower (~1.25 fold). When the results were represented as the number of condensations per unit original surface area, by correcting for the applied strain, the 0–20hr strain condition was found to be statistically different from the non-strained and the later-strained conditions (Fig 3C).

### Differentiation

**Fig 4:**
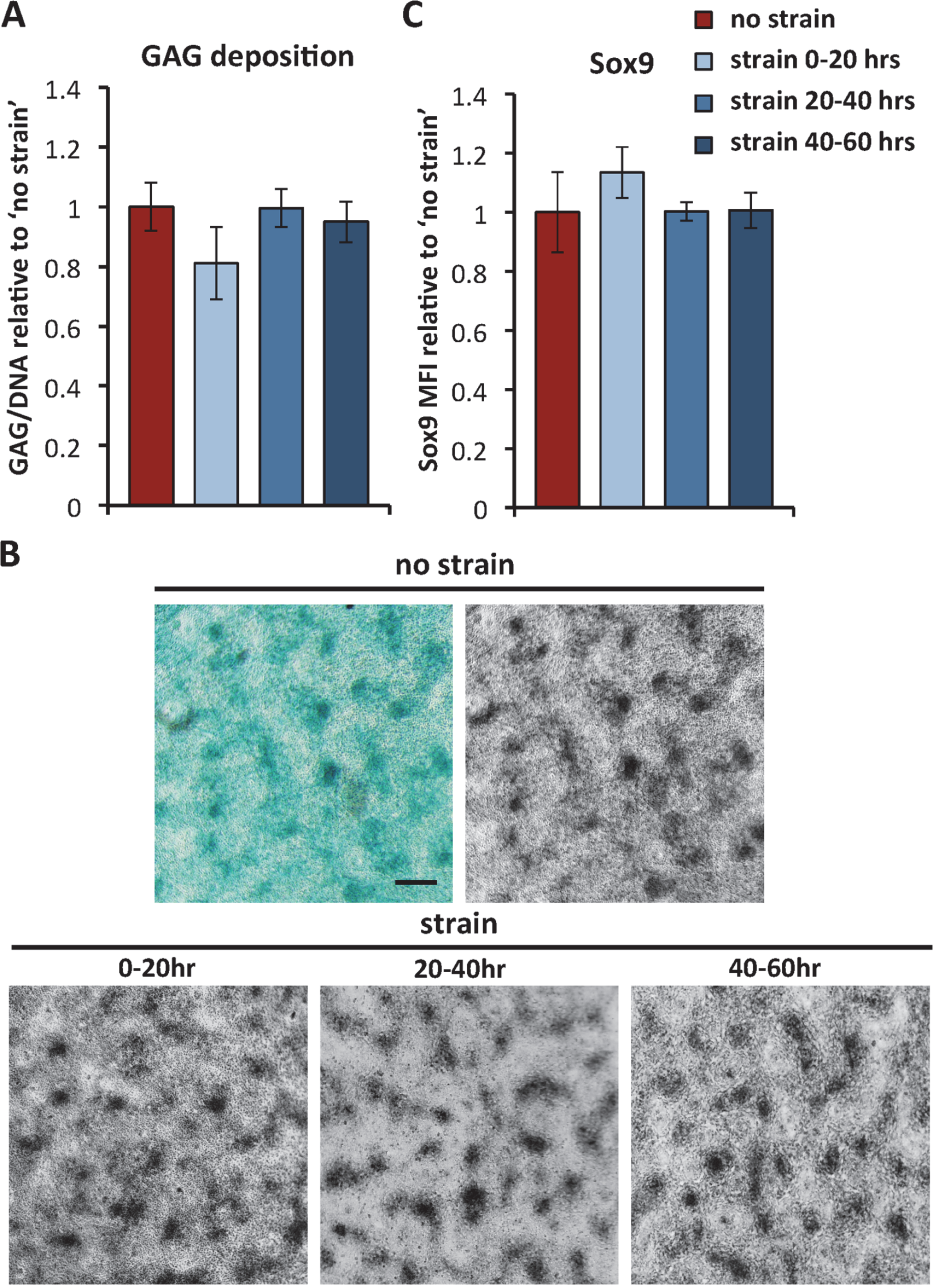
Chondrogenic differentiation. **(A)** Glycosaminoglycan (GAG) deposition is quantified in whole samples after 60 hours in culture, and normalized to DNA content. Data are normalized to the non-strained condition and represent means ± standard deviations, n≥5. **(B)** Samples cultured for 60hrs are stained with Alcian Blue to visualize glycosaminoglycan deposition. Color images are converted to gray scale, and an example of this conversion is shown for the non-strained condition. Scale bar is 200μm. **(C)** The mean fluorescent intensity (MFI) of cells stained for Sox9 was quantified using flow cytometry as a measure of the relative expression of Sox9 after 60 hrs. Data are normalized to the non-strained condition and represent means ± standard deviations, n≥4.

Lastly, it was investigated how growth-mimicking strain affects differentiation in micromass culture. As a measure of early chondrogenic differentiation, the deposition of glycosaminoglycans was measured and normalized to DNA content. No significant differences were observed between the non-strained and the various strained conditions (Fig 4A). Histological staining of GAG deposition resulted in similar staining patterns (Fig 4B). Color images were converted to gray scale, as shown for the non-strained condition, to allow for easier comparison between samples from different experiments. For all conditions, enhanced staining was observed in the condensations, as compared to the regions in between the condensations. Additionally, the expression of the transcription factor Sox9 was measured, as this is known to be an important player in the onset of chondrogenic differentiation, especially during embryonic development [26]. In line with the GAG deposition, no significant differences were found in Sox9 expression between the different conditions (Fig 4C).

## Discussion

Embryonic skeletal precursor cells in micromass were subjected to uniaxial continuous strain at different times to study the effect of growth-mimicking strain on the processes of proliferation, mesenchymal condensation and early chondrogenic differentiation in a highly controlled manner *in vitro*. Significant differences were found in the density of condensations, depending on the timing of strain. However, we found that proliferation, condensations morphology, and the extent of chondrogenic differentiation were not affected by continuous strain at any time during culture.

Our findings suggest that the process of condensation is sensitive to growth-mimicking strain only in the first 20hrs of culture. The density of condensations in cultures that were strained from 20–40hrs and from 40–60hrs were found to be significantly lower than those in cultures that were not strained at all, or strained from the start of culture (0–20hrs) (Fig 3B). Possibly, the same overall number of condensations was initiated in the 20–40hrs and 40–60hrs strain conditions before the strain was started, as compared to the non-strained condition. When the cultures were then strained, the total surface area increased while the number of condensations stayed constant, resulting in a decreased density of condensations. Indeed, when the results are corrected for the 25% strain, representing the number of condensations per unit original surface area, the numbers for the 20–40hrs and 40–60hrs are similar to the non-strained condition (Fig 3C). However, when cultures are strained right from the start of culture, from 0–20hrs, the density of condensations is similar to the non-strained condition (Fig 3B), and thus significantly higher when corrected for the total strain (Fig 3C). In previous studies, early signs of condensations in micromass were observed at 24hrs [23], indicating that condensations are initiated before or around that time. Our results might indicate that in the first 20hrs, while the condensations are initiated, the culture is sensitive to the applied strain, leading to an increased number of condensations. This would correspond to a phenomenon described in marine anglefish, whose skin patterns do not simply enlarge during growth, but continuously rearrange to maintain the spaces between the lines [27]. Another possible explanation is that the formation of condensations might only be initiated towards the end of the 0–20hr strain period, when the surface area was already increased to almost its final size, resulting in a comparable density of condensations as on the non-strained condition (Fig 3B). Further studies into the mechanisms of condensation and the application of additional strain regimes will provide better insight into the exact role of growth-mimicking strain on the spacing and patterning of condensations.

The results of this study further show that there is no significant effect of 20hrs of continuous strain on the proliferation, condensation morphology and chondrogenic differentiation of embryonic skeletal precursor cells in micromass culture. In line with the observation that adult bone cell activation decreases with decreasing fluid shear stress rate [28], this may indicate that these cells are simply not sensitive to low strain rates, at least at this stage in the lineage commitment under the tested conditions. However, other studies have reported an impact of mechanical loading on embryonic skeletal precursor cells in micromass culture [20,21]. Widely varying loading regimes were used in those studies, which may underlie the different or even opposite outcomes. Although embryonic skeletal progenitor cells were used in the aforementioned studies, their loading regimes were designed to mimic loading of adult cartilage structures and the chondrogenic commitment of embryonic versus adult MSCs takes place in highly distinctive mechanical environments. In the present study, we applied a slow continuous strain to mimic growth-induced strain in the developing embryo. It is important to note that the matrix composition and rigidity of the micromass culture used here might compromise the cells’ ability to sense and respond to the mechanical loading applied to the underlying substrate. More sophisticated cell culture models may be required to address this possibility and to further investigate the role of mechanical loading. Further studies along these lines will provide greater insight into the role of mechanics in early skeletal development and guide skeletal tissue engineering strategies.
